# Low-loss optical waveguides made with a high-loss material

**DOI:** 10.1038/s41377-020-00454-w

**Published:** 2021-01-12

**Authors:** Darius Urbonas, Rainer F. Mahrt, Thilo Stöferle

**Affiliations:** IBM Research Europe—Zurich, Säumerstrasse 4, 8803 Rüschlikon, Switzerland

**Keywords:** Optoelectronic devices and components, Sub-wavelength optics, Silicon photonics, Nanophotonics and plasmonics

## Abstract

For guiding light on a chip, it has been pivotal to use materials and process flows that allow low absorption and scattering. Based on subwavelength gratings, here, we show that it is possible to create broadband, multimode waveguides with very low propagation losses despite using a strongly absorbing material. We perform rigorous coupled-wave analysis and finite-difference time-domain simulations of integrated waveguides that consist of pairs of integrated high-index-contrast gratings. To showcase this concept, we demonstrate guiding of visible light in the wavelength range of 550–650 nm with losses down to 6 dB/cm using silicon gratings that have a material absorption of 13,000 dB/cm at this wavelength and are fabricated with standard silicon photonics technology. This approach allows us to overcome traditional limits of the various established photonics technology platforms with respect to their suitable spectral range and, furthermore, to mitigate situations where absorbing materials, such as highly doped semiconductors, cannot be avoided because of the need for electrical driving, for example, for amplifiers, lasers and modulators.

## Introduction

Guiding of light is fundamental to optical communication and integrated photonic circuits. To confine the propagating electromagnetic waves and guide them with low loss, the use of select dielectrics and semiconductors^1–3^ with excellent optical transparency is of utmost importance. However, for many devices, such as modulators^4^ and amplifiers^5^, strongly absorbing materials are needed for electrodes in the direct vicinity of the guided light to maximize the speed and efficiency^6^. Furthermore, the application-specific spectral range imposes restrictions on the possible material set and technology;^7^ for example, the common silicon photonics platform is not usable for visible light. For free space optics, the concept of subwavelength gratings with high refractive index contrast was introduced to construct broadband, highly reflective infrared mirrors^8,9^. Here, we create on-chip waveguides from pairs of high-index-contrast silicon gratings for lateral confinement by reflection at grazing incidence and a thin conformal silicon oxynitride layer for vertical confinement. Using standard silicon photonics fabrication technology, we achieve multimode, broadband guiding with losses as low as 6 dB/cm in the wavelength range of 550–650 nm, despite the silicon material absorption of the lateral guiding structure being 13,000 dB/cm. This concept will not only allow the use of absorbing electrodes in photonic circuits but also, more generally, open up new application realms for current photonics technology platforms used for communication, biosensing^10^ or photonic quantum computing^11,12^, such as silicon, indium phosphide and gallium arsenide, beyond their inherent spectral limitations.

Optical waveguides are a key element in all photonic integrated circuits^13^ because they interconnect different components and are intrinsic for devices such as Mach–Zehnder modulators^14^, semiconductor optical amplifiers^15^ and travelling wave resonators^16,17^. Most waveguides are based on total internal reflection (TIR) and consist of a high-refractive-index core surrounded by a low-refractive-index cladding. To achieve low-loss light confinement, tremendous efforts have been made over decades to reduce absorption and scattering^18–23^ within the core and cladding materials, resulting in a narrow set of suitable elaborate processing technologies and materials^1,24^. Waveguides using reflective boundaries instead of TIR have been mainly investigated in the context of metal-clad^25,26^ or photonic crystal waveguides^27,28^. However, for metal-clad structures, the high losses in strong light confinement and exciton quenching in nearby active materials pose issues for their use in integrated photonics. Moreover, photonic crystal waveguides do not show very low losses and have not attained wide adoption because of the challenging fabrication and restrictive set of suitable materials. More recently, planar surfaces with high reflectivity over a broad spectral range were created by using linear gratings made from dielectric or semiconducting materials with high refractive index contrast compared to their surroundings^8,29^. These high-contrast gratings (HCGs) with a period smaller than the optical wavelength harness a special reflection mechanism^30,31^ based on the constructive and destructive interference of two modes supported by the grating that occurs between the diffraction and deep subwavelength grating regimes^32^. While a number of concepts to use pairs of HCGs for waveguiding have been developed^33^, only a few have been actually realized in experiments^34^ due to the extremely demanding fabrication. Planar waveguides with low loss that are fully compatible with existing photonic circuit fabrication techniques are missing. Furthermore, it has been overlooked that such waveguides exhibit the potential for uniquely low sensitivity to material absorption inside the gratings^35^.

**Fig. 1:**
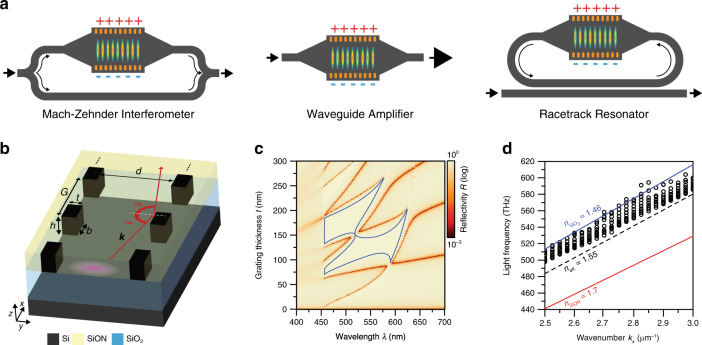
Light reflection from an HCG and waveguiding. **a** Conceptual illustration of how HCGs could be integrated into a set of devices as absorptive electrodes to drive the respective active region. Here, the light (displayed as arrows) travels in standard passive waveguides and enters and exits the HCG sections (orange gratings) with the active material in between through adiabatic mode conversion tapers. **b** Basic geometry of an HCG waveguide with grating thickness *t*, period *G*, fill factor *η* = *b*/*G* and waveguide width *d*. Light with wavevector ***k*** at high incidence angle *θ* is reflected at the grating (red arrows). The pink area illustrates the cross-section of the HCG waveguide mode. **c** Reflectivity spectrum calculated with RCWA of a single HCG as the grating thickness *t* is varied while keeping other geometry parameters fixed at *η* = 60%, *G* = 135 nm and *θ* = 80°. Blue contour lines enclose the regime where reflectivity *R* > 99% holds, showing that the high reflectivity is broadband (>100 nm in wavelength at *t* = 135 nm) and resilient to small grating thickness variations (±15 nm at a *λ* = 550 nm wavelength). **d** 3D FDTD simulated waveguide dispersion for *E*_*z*_-polarized light with the geometry parameters *η* = 60%, *t* = 135 nm, *h* = 220 nm and *d* = 5000 nm. Black circles show the different modes supported by the HCG waveguide. The red and blue lines indicate the SiON and SiO_2_ light lines, respectively. The black dashed line corresponds to an effective index *n*_eff_ = 1.55

Here, we report HCG waveguides fabricated with standard silicon photonics technology using a design that minimizes the spatial overlap of the propagating modes with the grating material. Despite silicon being strongly absorbing in the visible wavelength region—it is widely used for photovoltaic systems—we demonstrate low-loss guiding over centimetre distances using a pair of silicon HCGs for lateral light confinement. In Fig. 1a, we conceptually illustrate how this approach could be applied in established device concepts to realize electrodes that confine and guide light while enabling charge injection into a region with an active material.

The basic geometry of light reflection at an HCG is sketched in Fig. 1b. To maximize the HCG effect, the ratio between the refractive indices of the grating *n*_high_ and the surroundings *n*_low_ should be as high as possible; in our case, we use silicon (Si, *n*_high_ = 4.1) and silicon oxynitride (SiON, *n*_low_ = 1.7), respectively, at a wavelength of *λ* = 600 nm. Using rigorous coupled-wave analysis (RCWA), we calculate the reflectivity of a single HCG at a high incidence angle of *θ* = 80° for electromagnetic waves with *E*_*z*_ linear polarization as a function of the HCG geometry (Fig. 1c and Supplementary Fig. 1a), revealing a spectrally broad reflectivity maximum. Furthermore, this reflectivity plateau is responsible for a large tolerance against deviations from the ideal grating geometry, thereby easing the requirements for the fabrication process. High reflectivity is maintained up to a *θ* = 75° incidence angle (see Supplementary Fig. 1b), corresponding to an effective numerical aperture for the waveguide of up to NA = 0.5. For the other linear polarization (*E*_*y*_), the effect is much less pronounced (Supplementary Fig. 2). When combining two parallel HCGs with a 5 µm distance to form the lateral boundaries of a waveguide (Fig. 1b), additional vertical confinement is provided by TIR inside the SiON layer at the boundaries to the substrate (SiO_2_, $n_{{\mathrm{SiO}}_{2}}=1.46$) and the cladding (air, *n*_air_ = 1). Figure 1d displays the waveguide modal dispersion supporting multiple guided modes with an effective index *n*_eff_ between 1.46 and 1.55, obtained by three-dimensional finite-difference time-domain (3D FDTD) simulations.

**Fig. 2:**
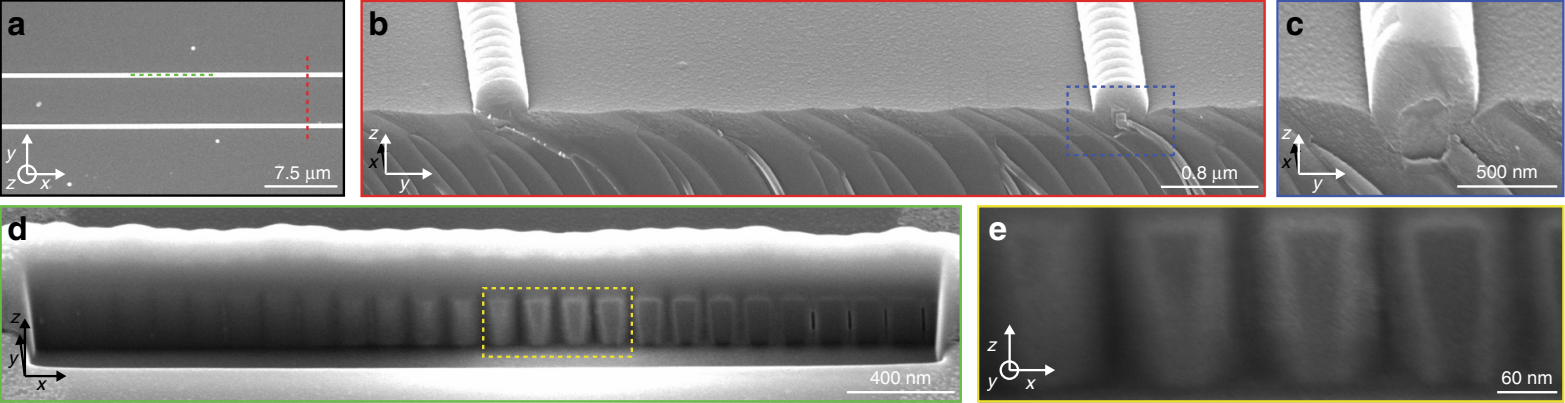
Fabricated HCG waveguides. **a** Scanning electron microscopy (SEM) image of the fabricated HCG waveguide from the top. **b** SEM tilt view of a cleaved waveguide facet, indicated by the red dashed line in (**a**). **c** SEM close-up image of a grating element indicated by the blue dashed region in (**b**). **d** SEM image of a cross-section cut by focused ion beam milling through the grating, indicated by the green dashed line in (**a**). **e** SEM close-up image of four grating elements, indicated by the yellow dashed region in (**d**)

We fabricate HCG waveguides using a state-of-the-art silicon-on-insulator (SOI) processing platform (Fig. 2 and see ‘Methods’). To assess the propagation loss, we realize waveguides of different lengths between 2 and 8 mm. For in- and out-coupling of light from/to optical fibres above the chip, we add micrometre-sized Si blocks at both the beginning and end of each waveguide to provide controlled vertical scattering from/to the waveguide without introducing pronounced measurement artefacts in the spectral range of interest (see Supplementary Fig. 3). Compared to the idealized sketch (Fig. 1b), the actual fabricated devices show several geometrical artefacts due to the specific growth of SiON, such as bumps on top of the HCGs (Fig. 2c), voids between the HCG elements (Fig. 2e) and tapering of the HCG elements (Fig. 2e). Owing to the robustness of the HCG design, these factors do not significantly affect the performance, but can be accounted for as an altered effective index in the simulations.

**Fig. 3:**
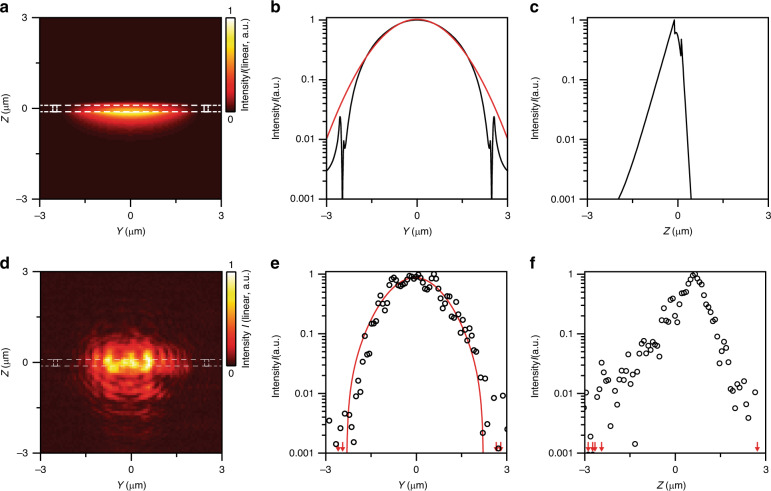
Waveguide mode cross-section. **a** 3D FDTD simulation obtained under multimode excitation of the waveguide. **b** Mode cross-section of (**a**) at *Z* = 0, where the black line is the simulated data and the red line is a Gaussian fit (FWHM = 2.9 µm). **c** Mode cross-section of (**a**) at *Y* = 0, where the black line is the simulated data. **d** Experimental far-field image of guided light emitted from a cleaved waveguide facet. **e** Cross-section of (**d**) at *Z* = 0, where the black circles are the measured data and the red line is a Gaussian fit (FWHM = 1.9 µm). **f** Cross-section of (**d**) at *Y* = 0, where the black circles are the measured data. Red arrows in (**e**, **f**) indicate data points outside the plot window. White dashed lines in (**a**, **d**) show the outline of the waveguide structure as defined in Fig. 1b

First, we measure the waveguide mode profile and compare it with the simulated profile. Figure 3a shows the 3D FDTD simulated guided mode profile (*E*_*z*_ polarization; see Supplementary Fig. 4 for *E*_*y*_ polarization), taking into account the actual fabricated geometry. The cross-section of the guided light deviates from a perfect Gaussian owing to the multimode nature of the waveguide (Fig. 3b, c). The measured mode profile obtained via far-field imaging from a cleaved waveguide facet (Fig. 3d–f) agrees well with the simulation, considering the optical resolution of the setup and variations in the in-coupling conditions due to the multimode guiding. From the simulations, we find that the integrated optical field inside the silicon HCG elements is only 4 × 10^–4^ of the total field intensity of the waveguide mode (Supplementary Fig. 5); therefore, this small overlap causes only low guiding loss despite high absorption in the silicon. More quantitatively, from the simulated overlap and the silicon absorption of ~3880 cm^−1^ at *λ* = 600 nm, we expect ~6.7 dB/cm of the waveguide loss to be attributed to the material absorption in the silicon elements, whereas any excess loss is most likely related to lateral leakage, scattering or absorption in other materials.

**Fig. 4:**
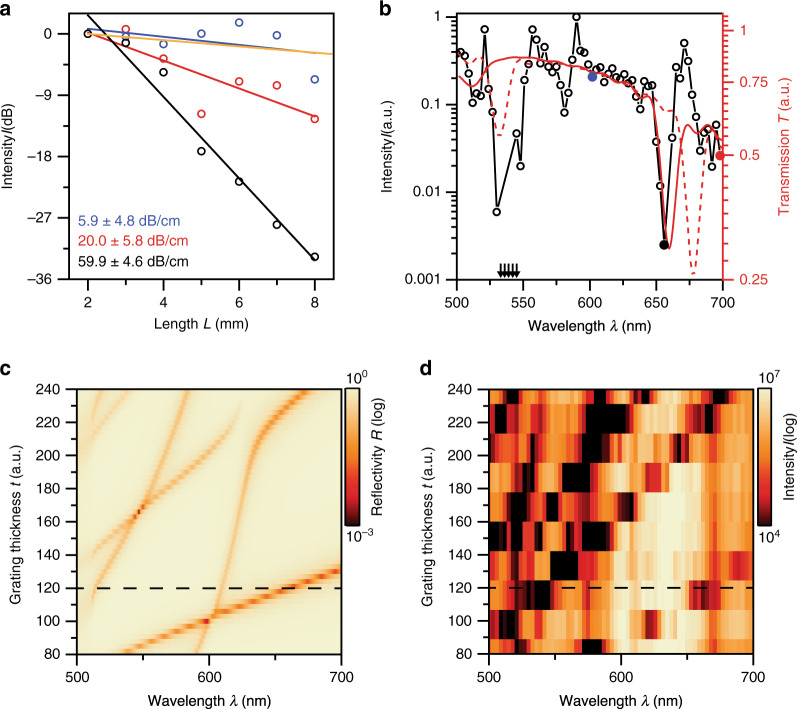
Propagation loss and transmission spectrum. **a** Transmitted intensity as a function of waveguide length for different wavelengths, which are indicated in (**b**) by dots of the respective colour. Coloured circles are data points (each from a different waveguide device with the indicated length and *t* = 120 nm) measured for *E*_*z*_ light polarization. Solid lines indicate linear fits to obtain the waveguide propagation loss. 3D FDTD simulations with excitation centred at approximately *λ* = 602 nm are shown as the orange line (see also Supplementary Fig. 5c). **b** Transmission spectrum through a 7-mm-long waveguide device with the experimental data shown as circles. Red dashed and solid lines are the 3D FDTD simulated spectra where gaps between HCG elements are filled with either air (*n*_air_ = 1) or SiON (*n*_SiON_ = 1.7), respectively. Coloured solid points show the wavelengths of the correspondingly coloured data in (**a**). Arrows indicate data points outside the displayed range. **c** Calculated reflectivity spectra of a single HCG obtained from RCWA for a *θ* = 85° incidence angle where the grating thickness *t* is varied. To achieve better agreement with the experiment, the full complex refractive index dispersion of Si is included, and the fill factor *η* = 65% and SiON refractive index *n*_SiON_ = 1.9 are slightly altered within the fabrication uncertainty boundaries. **d** Measured transmission spectra of 7-mm-long HCG waveguides with *t* = 80–240 nm (10 different geometries). Dashed black lines in (**c**, **d**) indicate the waveguide geometry from which the experimental data in (**a**, **b**) are obtained

Next, we measure the transmission loss and spectrum of the light propagating through the HCG waveguide and compare it to simulations. Figure 4a displays the propagation losses of the HCG waveguides at different wavelengths (*E*_*z*_ polarization; see Supplementary Fig. 5 for *E*_*y*_), obtained by measuring the transmission through waveguides of different lengths (see ‘Methods’). The blue data show the intensity *I* versus propagation distance for a mode near *λ* = 600 nm, which is guided by the HCG effect and exhibits 5.9 ± 4.8 dB/cm losses (derived from a linear fit, blue solid line), in agreement with 3D FDTD simulations of the corresponding device (orange line). Outside the HCG regime, leaky modes that are partially guided by Fresnel reflection at the HCG experience moderate losses of 20 dB/cm (red data). At the HCG resonances, where the mode overlaps much stronger with the absorbing Si HCGs, the losses reach 60 dB/cm (black data).

The transmission spectrum after 7 mm of propagation is shown in Fig. 4b, revealing a broad spectral range with losses below 10 dB/cm between the HCG resonances at 540 and 660 nm, in good agreement with 3D FDTD simulations (red lines). The offset in wavelength is caused by the effective index deviations stemming from voids between Si blocks and the overgrown SiON layer on top of the HCG. The reflectivity of a single HCG calculated with RCWA including these fabrication artefacts (Fig. 4c) agrees well with the measured losses from HCG waveguides when the thickness *t* of the HCGs was varied (Fig. 4d for *E*_*z*_ polarization; see Supplementary Fig. 6 for *E*_*y*_ polarization and Supplementary Fig. 7, where the same data for *E*_*z*_ and *E*_*y*_ polarizations are plotted with the full dynamic range). The key identifying features are the resonance dips that agree well between the simulated and experimental data.

To explore the intrinsic limits of the HCG waveguide concept above, we use high-quality SiON as a vertical guiding layer. In application contexts such as hybrid organic–inorganic modulators^6^ or creating amplifiers or circuits with organic all-optical transistors^36^, a polymer will constitute the guiding layer instead. Straightforwardly, spin-coating can be used for easy and cost-efficient fabrication, resulting in smooth guiding layers (see Supplementary Fig. 8). In such settings, depending on the optical properties of the polymer, the propagation losses might then become dominated by the absorption and scattering in the polymer and not by the silicon gratings.

While many interesting applications of HCG waveguides are already conceivable employing straight waveguides only (see Fig. 1a), a further important aspect for a more general integrated photonics platform is the realization of waveguide bends. From the high NA = 0.5 of the HCG waveguide (for >99% reflectivity), one can expect that bend radii >50 µm should, in principle, perform reasonably well. Therefore, we conduct a 3D FDTD calculation to simulate the transmission through an S-bend of two times 8° (see Supplementary Fig. 9). For a bend radius of 100 µm, we obtain a transmission loss on the order of 1 dB, caused mostly by losses after the bend from the scrambled modes. This demonstrates the general feasibility of guiding light around corners with HCG waveguides. We note, however, that optimization of the geometry (non-constant bend radius, blazed gratings, changing waveguide width, etc.) has the potential to further improve this result, allowing sharper bends with lower losses and reducing the mode mixing that leads to subsequent losses in the straight section following the bend.

Our experiments demonstrate that, counterintuitively, even materials wherein light is absorbed within a few micrometres can be used to guide light with low losses over a centimetre distance. Moreover, an important feature for high-yield manufacturing is the robustness of the HCG waveguide performance to fabrication inaccuracies due to the broadband guiding and wide geometry parameter window for highly reflective gratings. This paves the way for using silicon photonics with visible light and allows optoelectronic devices and circuits from a variety of different fields, ranging from biosensing to quantum technologies, to benefit from this versatile light-guiding platform, enabling access to an unprecedented wide choice of materials. Furthermore, it enables a novel way to integrate into established device concepts a highly doped material that is strongly absorbing in direct contact with an active material, acting as electrodes for efficient charge injection. It is conceivable to use adiabatic tapers to convert the mode between such driven HCG waveguide sections and other passive waveguides.

## Methods

### Simulations

RCWA is a semianalytical method where structures and fields are represented as a sum of spatial harmonics^37^. It is used to simulate scattering from periodic grating structures with a maximum of 11 in-plane (*x* and *y*) Fourier expansion orders, including the complex refractive index dispersion of Si.

Full 3D FDTD simulations are ab initio calculations of the temporal behaviour of electromagnetic waves in the structure. The waveguide dispersion relation in Fig. 1c is calculated with the Meep software package^38^. A calculation cell of size 1 × 59 × 96 (in units of *G*) is used with periodic boundary conditions including a phase factor in the grating direction and perfectly matched layers (PMLs) as effectively absorbing boundaries elsewhere. The grating period is discretized by 6 mesh points, and the complex material dispersion relation including the material absorption is taken into account. The structure is excited by a plane wave launched in the centre between both HCGs along the waveguide, exhibiting a temporal Gaussian envelope that yields a 270 nm spectral width at a 600 nm central wavelength. The guided mode fields and spectra are calculated with the Lumerical FDTD: 3D Electromagnetic Simulator^39^. A calculation cell of size 888 × 59 × 29 (in units of G) is used with absorbing boundary conditions (PMLs). The grating period is discretized by a variable mesh with 24 mesh points at the grating and 2.7 mesh points close to the PMLs, and the complex refractive index dispersion of Si is considered. The HCG waveguide is excited by coupling from a ridge waveguide with the same dimensions as the HCG waveguide having an eigenmode propagating in the waveguide direction, launching a pulse with a temporal Gaussian envelope yielding a 200-nm spectral width at a 600-nm central wavelength. The HCG waveguide mode profiles are captured after propagating through 785 grating unit cells, and the spectra are captured after 822 unit cells of propagation distance. The simulated propagation losses are extracted from the mode intensity drop along the waveguide at the centre of the computational cell.

### Fabrication

The waveguides are fabricated on SOI substrates consisting of a 2-μm-thick buried oxide layer and a 220-nm-thick Si layer. The structures are defined by electron-beam lithography with a 100 keV beam energy using hydrogen silsesquioxane as a negative tone resist. Pattern transfer is achieved in a two-step HBr chemistry-based inductively coupled reactive ion etch process followed by a development step. The 220-nm-thick SiON deposition is carried out using plasma-enhanced chemical vapour deposition at 300 °C.

### Characterization

The sample is mounted on an *XYZ* stage with nanometre position control. As a light source, we use a supercontinuum source (NKT SuperK EXTREME EXU-6 with a SuperK SELECT Vis 1× tuneable filter) operating at an 80 MHz repetition rate with an effective pulse duration of ~10 ps. To measure the transmission spectra, the source is coupled from a single-mode optical fibre that is positioned at a small angle with respect to the normal of the sample surface over the centre of the scattering block element belonging to a specific HCG waveguide (see Supplementary Fig. 3). The light from the HCG waveguide is then out-coupled after propagation by an identical element, collected by a long working distance objective with an effective focal length of *f* = 2 mm and numerical aperture NA = 0.5 (Mitutoyo ×100 M Plan Apo NIR) and projected onto a camera. For the transmission measurements, we integrate the intensity recorded by the camera over the spatial region where the out-coupling block is located. To image mode profiles, one facet of the HCG waveguide is cleaved and imaged using the same objective.

Scanning electron microscopy (SEM) images are acquired using a Phenom ProX electron microscope operated at 10 kV. Focused ion beam (FIB) cross-section cuts are acquired using a dual beam FIB (FEI Helios). Prior to SEM and FIB processing, the samples are covered with a 5 nm platinum (Pt) layer using a magnetron sputtering process to reduce charging.

## Supplementary information

Supplementary Figures

## Data Availability

The data that support the findings of this study are available from the corresponding authors upon reasonable request.
