# Supplementary material for: Low-loss optical waveguides made with a high-loss material
Source: Light Sci Appl. 2021 Jan 12;10:15. doi: 10.1038/s41377-020-00454-w (PMC7804948; doi:10.1038/s41377-020-00454-w)
Supplement: Supplementary file 1 — Supplementary Figures [file 41377_2020_454_MOESM1_ESM.docx]

**Supplementary Information**

**Low-loss optical waveguides made with a high-loss material**

Darius Urbonas, Rainer F. Mahrt, and Thilo Stöferle

*IBM Research Europe – Zurich, Säumerstrasse 4, CH-8803 Rüschlikon, Switzerland*

**Supplementary Fig. 1 | HCG reflectivity versus fill factor and incidence angle** **for *E_z_*-polarized light.** RCWA calculations of the reflectivity spectrum of a single HCG where blue contour lines enclose regimes with reflectivity *R* > 99%. **a**, Reflectivity spectra when the grating fill factor *η* is varied, keeping other geometrical parameters fixed at *t* = 135 nm, *G* = 135 nm and *θ* = 80°. **b**, Reflectivity spectra with the same geometrical parameters when the grating incidence angle *θ* is varied for fixed fill factor *η* = 60%.

**Supplementary Fig. 2 | HCG reflectivity for *E_y_*-polarized light.** RCWA calculations of the reflectivity spectrum of a single HCG where blue contour lines enclose regimes with reflectivity *R* > 99%. **a**, Reflectivity spectra when the grating thickness *t* is varied, keeping other geometrical parameters fixed at *η* = 60%, *G* = 135 nm and *θ* = 80°. **b**, Reflectivity spectra with the same geometrical parameters when the grating fill factor *η* is varied for fixed grating thickness *t* = 135 nm. **c**, Reflectivity spectra with the same geometrical parameters when the grating incidence angle *θ* is varied for fixed fill factor *η* = 60%.

**Supplementary Fig. 3 | Basic light in- and out-coupling element.** **a**, Schematic *XZ* cross section (*Y* = 0) of the scattering structure where the waveguide extends towards the left. The Si block (in black) is 2 µm wide, 10 µm long across the waveguide in *Y* direction and 220 nm high. **b**, 3D FDTD simulated *E_x_* field component for an *E_y_*-polarized mode (incoming from the left, red arrow in **a**). **c**, Same as **b** but showing the *E_y_* field component. **d**, Same as **b** but showing the *E_z_* field component. **e**, SEM top view of a fabricated scattering element from which the HCG waveguide extends to the right. **f**, 3D FDTD simulated *E_x_* field component for an *E_z_*-polarized mode (incoming from the left, red arrow in **a**). **g**, Same as **f** but showing the *E_y_* field component. **h**, Same as **f** but showing the *E_z_* field component. For all field plots we use a saturated colour scale to make the scattered part better visible because only little intensity is scattered vertically from the horizontally propagating mode.


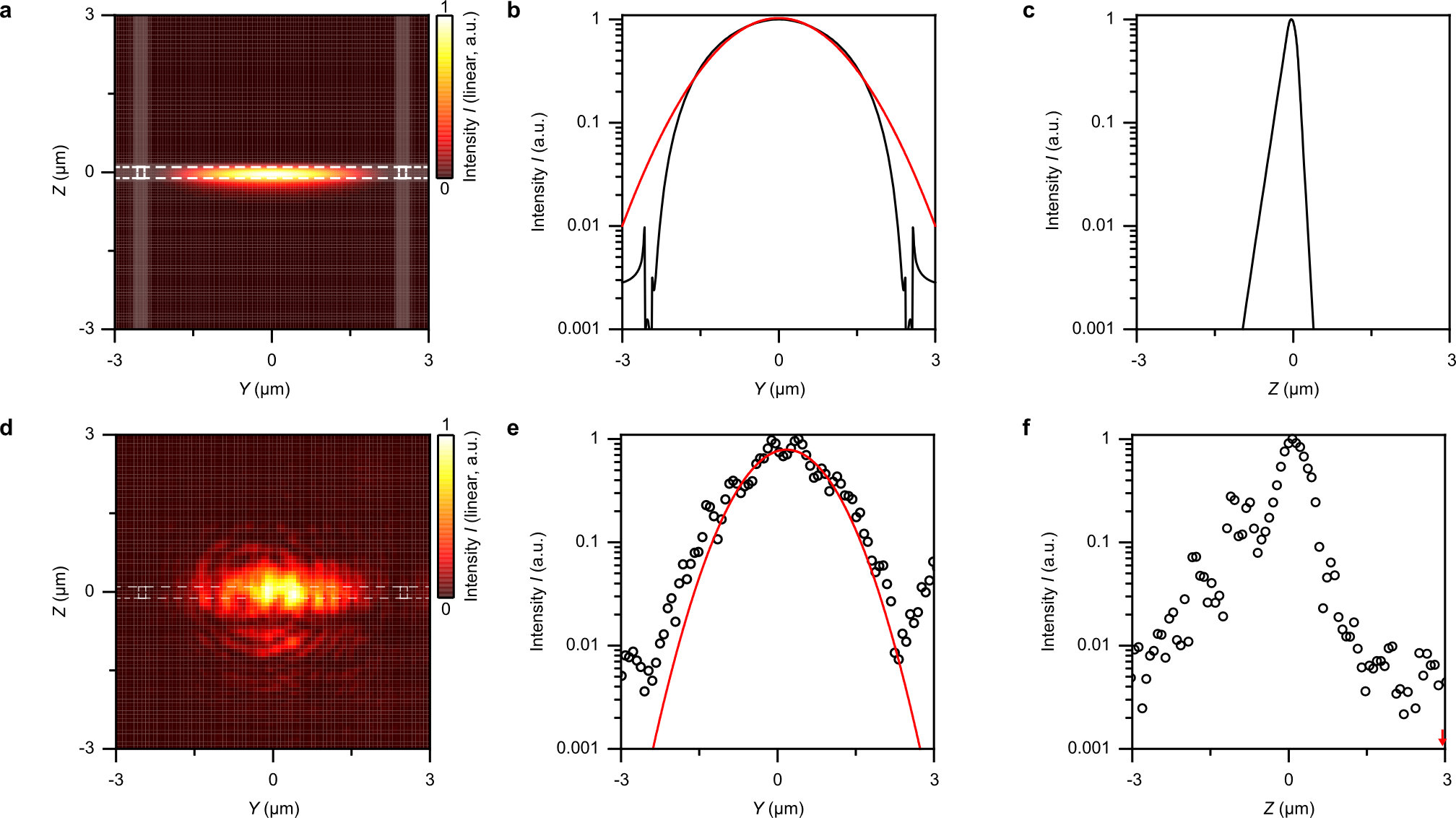


**Supplementary Fig. 4 | Waveguide mode cross section for *E_y_* light polarization.** **a**, 3D FDTD simulation of the guided mode obtained by multimode excitation of the waveguide. **b**, Mode cross section of **a** at *Z* = 0 where the black line represents the simulated data and the red line is a Gaussian fit (FWHM = 2.5 µm). **c**, Mode cross section of **a** at *Y* = 0 where the black line is the simulated data. **d**, Experimental far-field image of guided light emitted from a cleaved waveguide facet. **e**, Cross section of **d** at *Z* = 0 where black circles represent the measured data and the red line is a Gaussian fit (FWHM = 1.7 µm). **f**, Cross section of **d** at *Y* = 0 where the black circles display the measured data. Red arrow in **f** indicates data points outside the plot window. White dashed lines in **a** and **d** show the outline of the waveguide structure as defined in Fig. 1b.


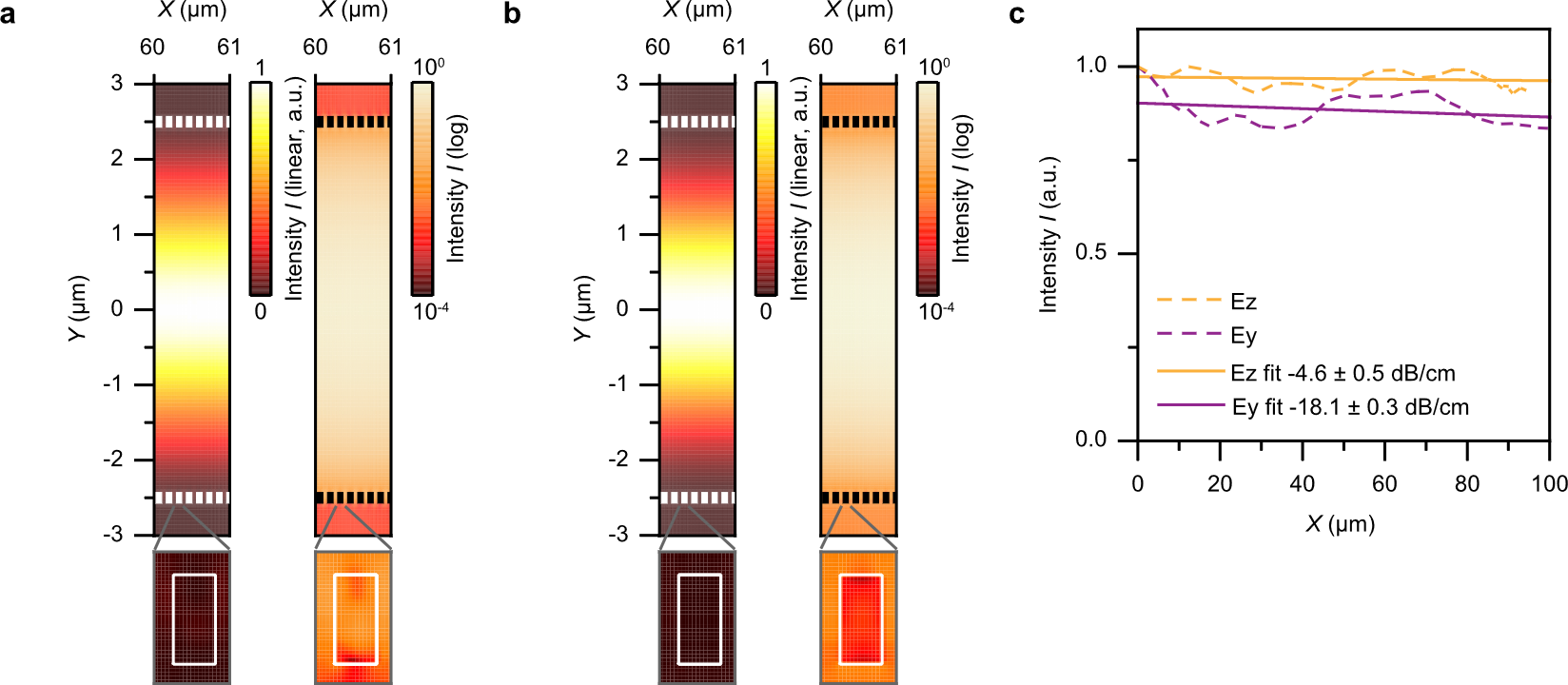


**Supplementary Fig. 5 |** **3D FDTD simulations of waveguide modes.** **a**, Top view (*XY* cross section at *Z* = 0) of the propagating field showing the electric field intensity in a short grating section for *E_z_* polarization with linear colour scale (left) and with logarithmic colour scale (right). White and black boxes indicate the Si grating blocks. A zoom-in to an individual block is shown below. **b**, Same for *E_y_* polarization. **c**, Simulated electric field intensity versus propagation distance for *E_z_*- and *E_y_*-polarized light. Dashed lines show the simulation, and solid lines depict respective linear fits to obtain the propagation loss. *X* = 0 corresponds to 20 µm after the source in order to have some mode cleaning and establishment of more stable propagating modes. Noise in the data is a numerical artifact because the simulated distance is too short to get rid of all leaky modes entirely.


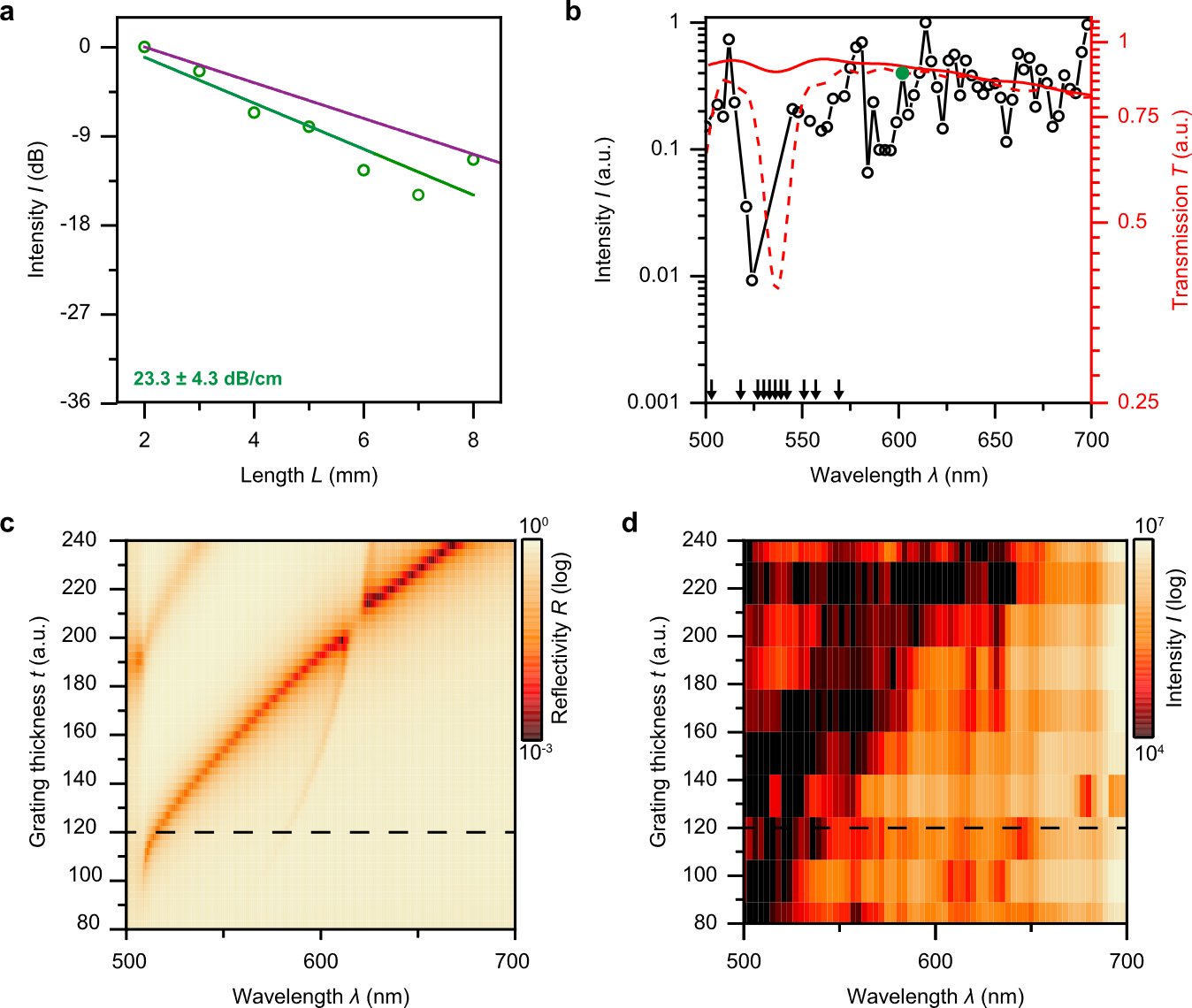


**Supplementary Fig. 6 | Propagation loss and transmission spectrum for *E_y_*-polarized light.** **a**, Transmitted intensity as a function of waveguide length for a specific wavelength indicated in **b** by a dot of the respective colour. Coloured circles are data points (each from a different waveguide device with the indicated length and with *t* = 120 nm) measured for *E_y_* light polarization. Solid lines indicate linear fits to obtain the waveguide propagation loss. 3D FDTD simulations with excitation centered around λ = 602 nm are shown as violet line. **b**, Transmission spectrum through a 7 mm-long waveguide device with the experimental data shown as circles. Red dashed and solid lines are the 3D FDTD simulated spectra where gaps between HCG elements are filled either with air (*n* = 1) or SiON (*n*_SiON_ = 1.7), respectively. Green solid disc shows the wavelength of the correspondingly coloured data in **a**. Arrows indicate negative data points due to normalization and noise. **c**, Calculated reflectivity spectra of single HCG obtained from RCWA for *θ* = 85° incidence angle and period *G* = 135 nm where the grating thickness *t* is varied. To achieve better agreement with the experiment, the full complex refractive index dispersion of Si is included and the fill factor η = 65% and SiON refractive index *n*_SiON_ = 1.9 were slightly altered within the fabrication uncertainty boundaries. **d**, Measured transmission spectra of 7 mm-long HCG waveguides having different *t*. Dashed black lines in **c** and **d** indicate the waveguide geometry from which the data in **b** is obtained.


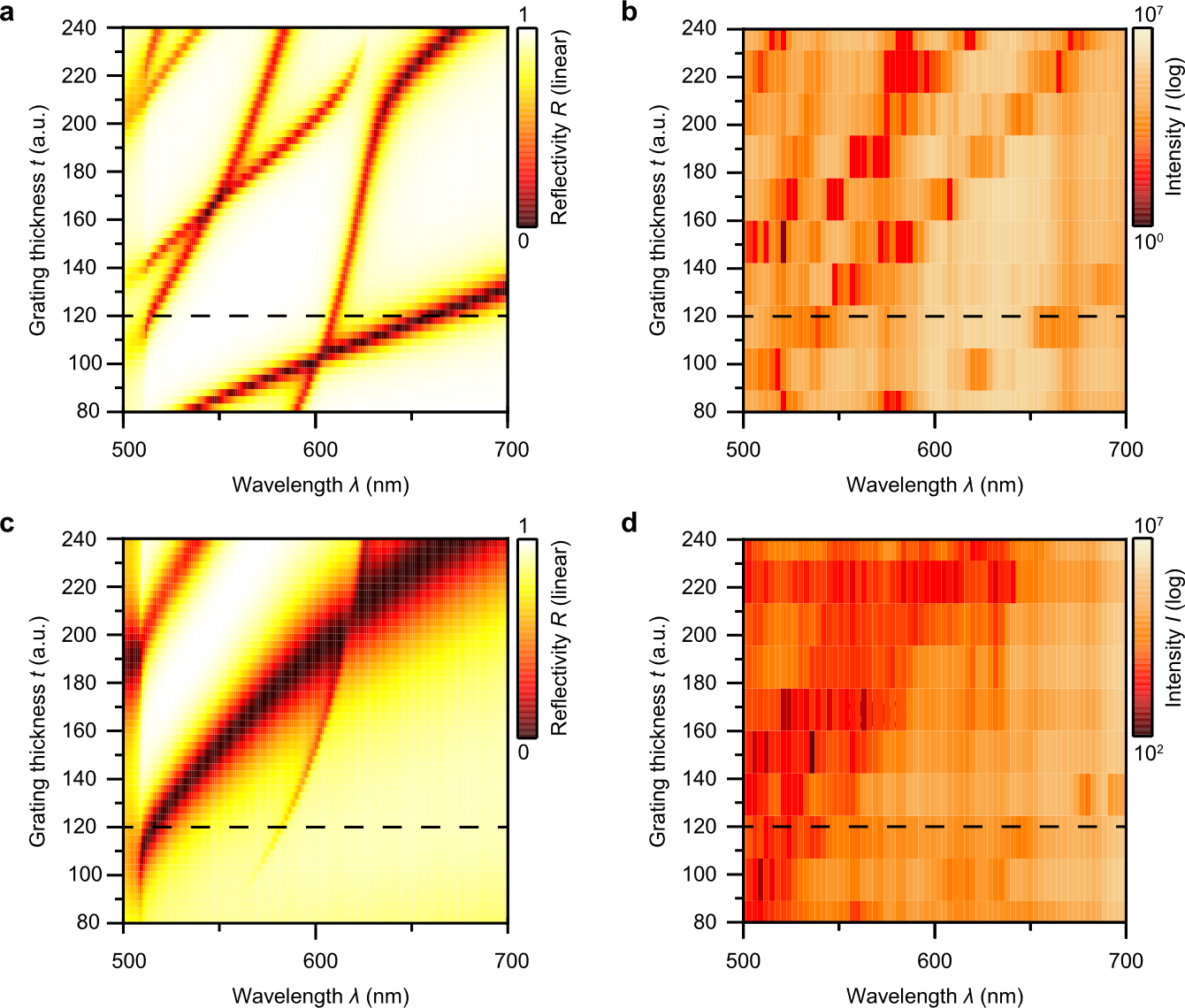


**Supplementary Fig. 7 | Optical properties of HCG waveguides for *E_z_* and *E_y_* polarizations plotted with full data dynamic range.** In Supplementary Fig. 6c,d and Fig. 4c,d logarithmic colour scales with some truncated data range are used to enhance the contrast of the line features in the data. **a**, Same data as Fig. 4c but shown in linear colour scale. **b**, Same data as Fig. 4d but shown with full data range without saturation. **c**, Same data as Supplementary Fig. 6c but shown in linear colour scale. **d**, Same data as Supplementary Fig. 6d but shown with full data range without saturation.


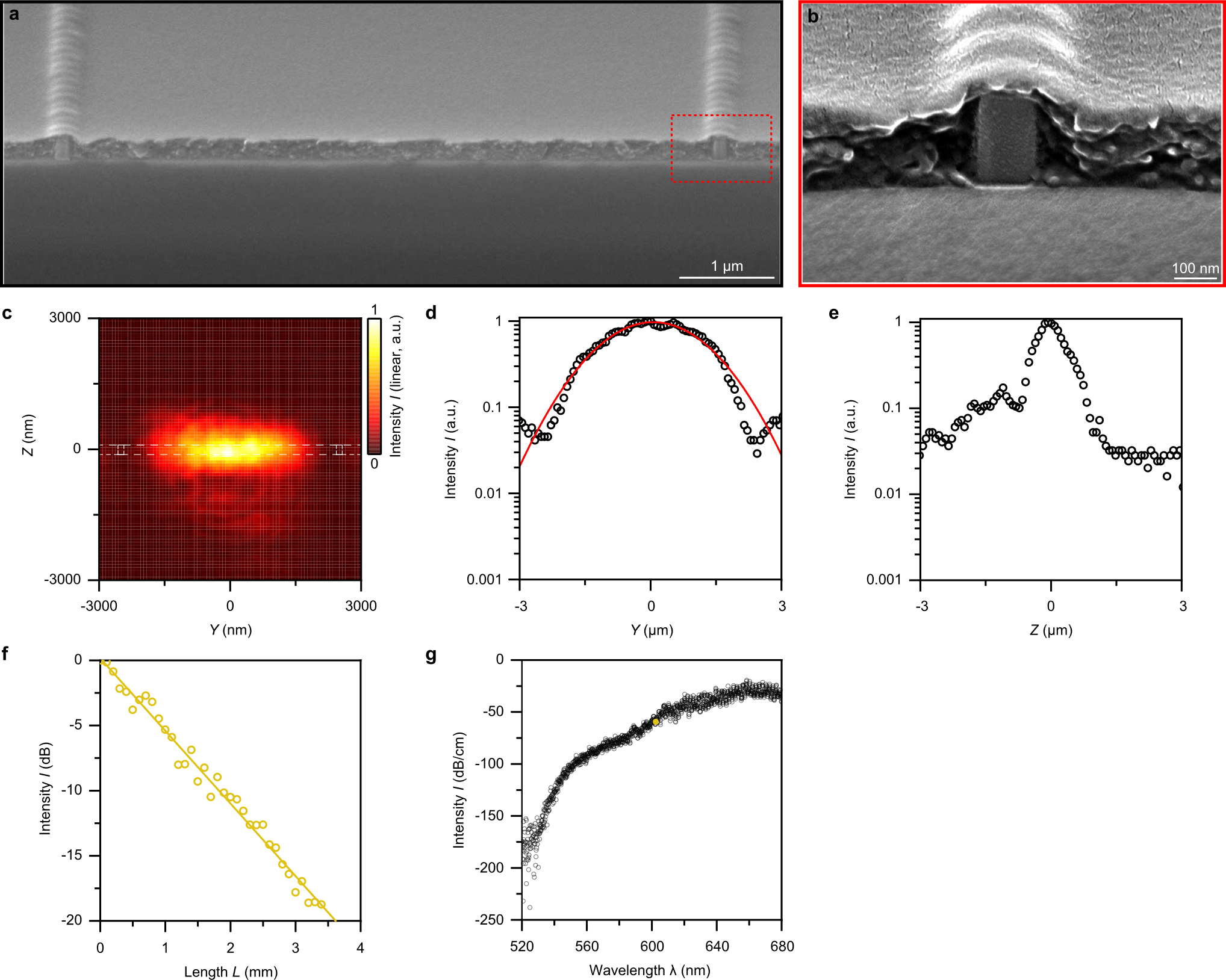


**Supplementary Fig. 8 | Properties of HCG waveguides with polymer layer.** In these devices the passive SiON layer is replaced with an emissive conjugated polymer (methylsubstituted ladder-type poly-[paraphenylene], MeLPPP), deposited by spin-coating. **a**, SEM tilt view of a cleaved waveguide facet. **b**, SEM close-up image of a grating element indicated as red dashed region in **a**. **c**, Experimental far-field image of guided light emitted from a cleaved waveguide facet. **d**, Cross section of **c** at *Z* = 0. The black circles represent the measured data and the red line is a Gaussian fit (FWHM = 2.6 µm). **e**, Cross section of **c** at *Y* = 0 in which the black circles display the measured data. White dashed lines in **c** show the outline of the waveguide structure as defined in Fig. 1b. **f**, By exciting the polymer locally, it can serve as “internal light source” instead of coupling via a silicon scattering block into the HCG waveguide. The bright, spectrally broad emission of MeLPPP is scattered by the HCGs, allowing to exploit the small loss to monitor directly the fall-off of the guided light. Here, the scattered intensity is shown as a function of waveguide length for a specific wavelength, indicated in **g** by a dot of the respective colour. Coloured circles are data points, each from a different waveguide device with the indicated length and with *t* = 160 nm, *η* = 60%, *h* = 220 nm, *G* = 135 nm and *d* = 5 µm. Solid line indicates a linear fit to obtain the waveguide propagation loss, resulting in 56 dB/cm. **g**, Scattered spectrum after propagation through a 3 mm-long waveguide device with the experimental data shown as circles. The spectral shape confirms that the relatively high losses are due to an absorption tail and/or scattering.


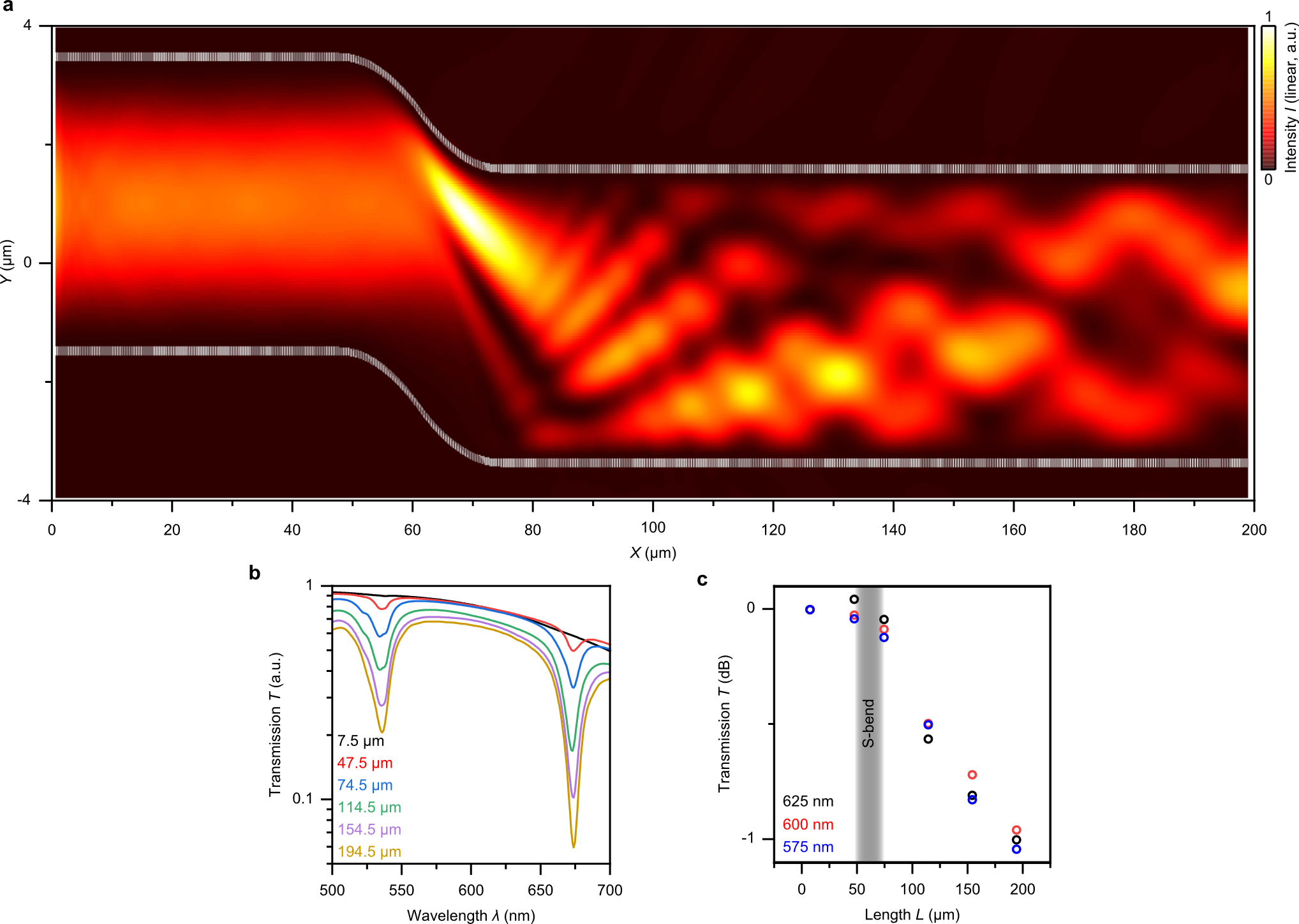


**Supplementary Fig. 9 | S-bend with HCG waveguides.** 3D FDTD simulations of an S-shaped bend with bend radius of 100 µm and a bend angle of two times 8 degrees. **a**, Simulated optical field intensity where a single mode is launched from the left. The S-bend induces excitation of several other modes that are guided in the subsequent straight section. Note that the aspect ratio of the displayed figure does not correspond to the simulated one as the vertical axis is plotted stretched by a factor of 10 to make the transversal mode structure better visible. White boxes indicate the Si grating blocks. **b**, Transmitted spectra at several positions along the waveguide, before, within and after the bend. **c**, Intensity versus distance from the left edge of the simulation cell for several wavelengths. Whereas the losses directly inside the bends are quite low (on the order of 0.1 dB), the largest losses occur after the S-bend due to the mode-mixing into leaky modes, reaching about 1 dB within the simulation cell.
